# SARS-CoV-2 Incidence in K–12 School Districts with Mask-Required Versus Mask-Optional Policies — Arkansas, August–October 2021

**DOI:** 10.15585/mmwr.mm7110e1

**Published:** 2022-03-11

**Authors:** Catherine V. Donovan, Charles Rose, Kanna N. Lewis, Kristyn Vang, Nichole Stanley, Michael Motley, Clare C. Brown, Franklin John Gray, Joseph W. Thompson, Benjamin C. Amick, Mark L. Williams, Ebony Thomas, John Neatherlin, Namvar Zohoori, Austin Porter, Mike Cima

**Affiliations:** ^1^CDC COVID-19 Emergency Response Team; ^2^University of Arkansas for Medical Sciences, Little Rock, Arkansas; ^3^Arkansas Department of Health; ^4^Arkansas Center for Health Improvement, Little Rock, Arkansas.

Masks are effective at limiting transmission of SARS-CoV-2, the virus that causes COVID-19 (*1*), but the impact of policies requiring masks in school settings has not been widely evaluated (*2*–*4*). During fall 2021, some school districts in Arkansas implemented policies requiring masks for students in kindergarten through grade 12 (K–12). To identify any association between mask policies and COVID-19 incidence, weekly school-associated COVID-19 incidence in school districts with full or partial mask requirements was compared with incidence in districts without mask requirements during August 23–October 16, 2021. Three analyses were performed: 1) incidence rate ratios (IRRs) were calculated comparing districts with full mask requirements (universal mask requirement for all students and staff members) or partial mask requirements (e.g., masks required in certain settings, among certain populations, or if specific criteria could not be met) with school districts with no mask requirement; 2) ratios of observed-to-expected numbers of cases, by district were calculated; and 3) incidence in districts that switched from no mask requirement to any mask requirement were compared before and after implementation of the mask policy. Mean weekly district-level attack rates were 92–359 per 100,000 persons in the community* and 137–745 per 100,000 among students and staff members; mean student and staff member vaccination coverage ranged from 13.5% to 18.6%. Multivariable adjusted IRRs, which included adjustment for vaccination coverage, indicated that districts with full mask requirements had 23% lower COVID-19 incidence among students and staff members compared with school districts with no mask requirements. Observed-to-expected ratios for full and partial mask policies were lower than ratios for districts with no mask policy but were slightly higher for districts with partial policies than for those with full mask policies. Among districts that switched from no mask requirement to any mask requirement (full or partial), incidence among students and staff members decreased by 479.7 per 100,000 (p<0.01) upon implementation of the mask policy. In areas with high COVID-19 community levels, masks are an important part of a multicomponent prevention strategy in K–12 settings (*5*).

COVID-19 incidence among K–12 students and staff members in Arkansas public school districts with different mask policies was investigated during August 23–October 16, 2021. Mask policies were defined as follows: 1) full (universal mask requirement for all students and staff members)^†^; 2) partial (masks required in certain settings [e.g., in classrooms but not in gym or music class], among certain populations [e.g., only certain grades, only students or staff members, or only unvaccinated persons], or if specific criteria [e.g., physical distancing ≥6 feet]) could not be met); and 3) none (masks not required in the school setting). Consistent with a Federal Order in place during the investigation period, all persons were required to wear masks while on school buses (*6*).

District-level data were compiled from the Arkansas Department of Health’s (ADH’s) COVID-19 surveillance database and immunization registry, Arkansas Center for Health Improvement’s mask policy database, and Arkansas Department of Education’s 2021–22 enrollment and 2019 free or reduced-cost school lunch databases. Four districts (2%) were excluded, including three serving special needs populations (blind, deaf, and incarcerated persons) and 1 year-round district.^§^

Data were analyzed using three different approaches: 1) IRRs and 95% CIs were used to compare districts with full or partial mask requirements to those with no mask requirements^¶^; 2) ratios of observed-to-expected numbers of cases were estimated by district (given the underlying weekly community COVID-19 incidence)** using negative binomial generalized estimating equation models with autoregressive correlation structure; and 3) associations between mask policy and COVID-19 incidence were estimated using a comparative interrupted time series model among students and staff members in a subset of 26 districts^††^ that began the school year without a mask requirement and subsequently transitioned to full or partial mask requirements.^§§^

District-level mask policies^¶¶^ (the exposure) were included in analyses based on the policy in place 1 week before school-associated COVID-19 incidence (the outcome) was measured.*** IRRs and ratios of observed-to-expected case numbers were adjusted for district-wide weekly COVID-19 non–school-associated (community) attack rates, district-wide weekly staff member and student vaccination coverage,^†††^ and the proportion of students receiving free or reduced-cost school lunches (as a proxy for socioeconomic status and educational disadvantage) (*7*). Weekly district-level vaccination coverage rates among students and staff members were calculated from the ADH immunization registry, which was matched to school district enrollment and staffing data based on name and date of birth. Sensitivity analyses were also conducted to evaluate the impact of varying lag times between the exposure and outcome and to investigate variations by grade level and vaccine eligibility.^§§§^ Statistical analyses were completed with SAS (version 9.4; SAS Institute). Statistical significance was defined as p<0.05. This project was reviewed and approved by ADH and CDC and was conducted consistent with applicable federal law and CDC policy.^¶¶¶^

During the investigation, statewide COVID-19 community transmission levels declined from substantial to moderate, and vaccination coverage increased.**** Among 233 included public school districts, 30%, 21%, and 48% had full, partial, or no mask policies, respectively, at baseline (August 22–28, 2021). Mean weekly district-level COVID-19 incidence among students and staff members was consistently higher than community incidence and decreased over time from 745 per 100,000 (August 29–September 4) to 137 per 100,000 (October 10–16); mean weekly school district level student and staff member vaccination coverage increased from 13.5% to 18.6% during the same period. COVID-19 incidence among students and staff members was 23% lower in districts with full mask policies than in districts with no mask policy (IRR = 0.77 [95% CI = 0.66–0.88]), 24% lower among staff members only (IRR = 0.76 [95% CI = 0.64–0.90]), and 23% lower among students only (IRR = 0.77 [95% CI = 0.66–0.89]) (Table). IRRs comparing districts with partial mask policies with those with no mask policy were not statistically significant (IRR = 0.88 [95% CI = 0.77–1.01] for students and staff members, 0.85 [95% CI = 0.71–1.02] for staff members only, and 0.89 [95% CI = 0.77–1.03] for students only).

**TABLE:** Estimated incidence rate ratios comparing weekly COVID-19 case incidence in kindergarten through grade 12 school districts with mask requirements to those without mask requirements — 233 school districts, Arkansas, August–October 2021

Group/School district mask policy	Adjusted IRR (95% CI)
Overall*	
None**^†^**	Ref.
Full**^†^**	0.77 (0.66–0.88)
Partial**^†^**	0.88 (0.77–1.01)
Among staff members*	
None	Ref.
Full	0.76 (0.64–0.90)
Partial	0.85 (0.71–1.02)
Among students*	
None	Ref.
Full	0.77 (0.66–0.89)
Partial	0.89 (0.77–1.03)
**Grades K–5^§^**	
None	Ref.
Full	0.78 (0.66–0.92)
Partial	0.88 (0.75–1.03)
**Grades 6–8^§^**	
None	Ref.
Full	0.69 (0.57–0.83)
Partial	0.83 (0.69–1.01)
**Grades 9–12^§^**	
None	Ref.
Full	0.68 (0.57–0.83)
Partial	0.79 (0.65–0.95)
School district student vaccination coverage, % (N)^¶,^**	
<10 (6–30)	Ref.
10–19 (29–101)	1.08 (0.80–1.46)
20–29 (72–75)	1.03 (0.77–1.39)
30–39 (22–69)	0.80 (0.58–1.11)
≥40 (8–54)	0.62 (0.44–0.87)

**Abbreviations:** IRR = incidence rate ratio; K = kindergarten; Ref. = reference group.

* Models were adjusted for week of school, COVID-19 incidence in the community during the preceding week, staff member and student vaccination rate in the previous week, and percentage of students in the district receiving free or reduced-cost lunch in 2019.

**^†^** Mask policies were defined as follows: 1) full (universal mask requirement for all students and staff members); 2) partial (masks required in certain settings [e.g., in classrooms but not in gym or music class], among certain populations [e.g., only certain grades, only students or staff members, or only unvaccinated persons], or if specific criteria [e.g., physical distancing >6 feet] could not be met); and 3) none (masks not required in the school setting).

**^§^** Models were adjusted for week of school, COVID-19 incidence in the community during the preceding week, and percentage of students in the district receiving free or reduced-cost lunch during 2019. Grade level–stratified models were not adjusted for vaccination coverage because students in grades K–5 were not eligible for vaccination, and estimates were stratified to allow for comparison across grade levels.

^¶^ Number of districts in each category varied over time, and N is shown as range over the course of the investigation.

** Among students in vaccine-eligible grades only (grades 7–12). Compared with <10% of district students vaccinated as the referent category. Models adjusted for mask policy, week of school, COVID-19 incidence in the community during the preceding week, and percentage of students in the district receiving free or reduced-cost lunch during 2019.

Ratios comparing observed-to-expected cases among students and staff members exceeded 1.0 for all groups (students only, staff members only, and combined students and staff members) and mask policies (Figure 1) (Supplementary Figure, https://stacks.cdc.gov/view/cdc/115046). The ratios of observed-to-expected cases for school districts with full mask policies for students only (1.50; 95% CI = 1.33–1.70); staff members only (1.69; 95% CI = 1.35–2.07) and combined students and staff members (1.52; 95% CI = 1.35–1.72) were lower than the ratios for no mask policy (students only: 2.06 [95% CI = 1.86–2.26]; staff members only: 2.44 [95% CI = 2.02–2.90] combined students and staff members: 2.10 [95% CI = 1.92–2.30]. Observed-to-expected ratios for school districts with partial mask policies were also lower than ratios for no mask policies, but slightly higher than those in districts with full mask policies.

**FIGURE 1:**
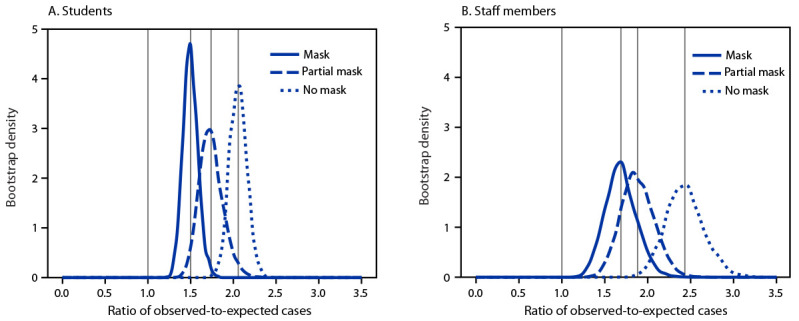
Mean estimates* of the ratio of observed school district cases to expected school district cases among students (A) and staff members (B), based on surrounding community incidence, by mask requirement status — 233 school districts, Arkansas, August–October 2021 * The mean estimates were calculated by drawing 5,000 random bootstrap samples from the dataset and averaging over all school districts with the same mask policy within each sample. The reference line at 1.0 implies that the school district incidence equals the community incidence. Vertical lines for each mask policy are the means for the 5,000 bootstrap samples and illustrate the difference of the group’s mean relative to the reference line. For example, the student and staff member mask group means are 1.50 and 1.69, which indicates that the mean incidences among students and staff members in school districts with mask requirements are 50% and 69% higher, respectively, than the mean incidence in their surrounding communities.

Among 26 districts that switched from no mask policy to any policy (full or partial) during the investigation, COVID-19 incidences for student and staff members were higher than those in the community during the period with no mask policy (estimated difference at baseline = 891.8 per 100,000, p<0.01). However, a week after implementation of a mask policy, the incidence among students and staff members decreased significantly (estimated point reduction in incidence = 479.7 per 100,000; p<0.01). Although the incidence among community members decreased at the same time (estimated point reduction in community incidence = 104.6 per 100,000, p<0.01), there was a significantly higher rate of reduction in incidence among students and staff members compared with that in community members (estimated difference in point reduction = 375.0 per 100,000; p<0.01) (Figure 2).

**FIGURE 2:**
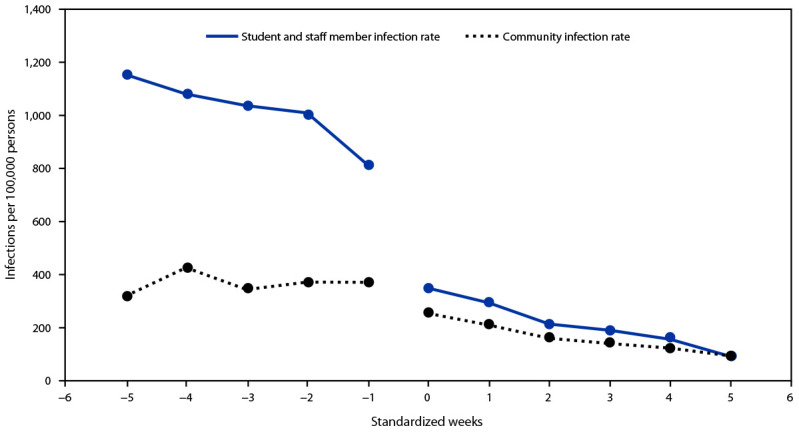
Student and staff member and community SARS-CoV-2 infection rates before and after* implementation of school mask requirement — 26 school districts, Arkansas, August–October 2021 * Weeks were standardized to align the time before (negative values) and after (positive values) the district changed from no mask requirement to partial or full mask requirement. Time zero indicates the week the policy changed from none to full or partial mask requirement, and the first weekly incidence under a mask requirement policy was measured during the following week. Upon implementation of the mask policy, the incidence among students and staff members decreased by 479.7 per 100,000. Incidence among community members decreased at the same time by 104.6 per 100,000, a difference of 375.0 per 100,000.

Sensitivity analyses demonstrated consistent findings. Analyses with 0-, 2-, and 3-week lag times were consistent with the initial analysis. Stratification by school level (grades K–5, 6–8, and 9–12) did not change the main results (Table). Adjusted student estimates stratified by vaccine-eligible (grades 7–12) and -ineligible (K–6) grade levels did not significantly differ from the unstratified estimates. Among vaccine eligible-grades, IRRs decreased with increasing student vaccination coverage. IRRs standardized to the surrounding community incidence were consistent with reported IRRs.

## Discussion

During August–October 2021, public school districts in Arkansas with full or partial mask requirements had lower incidences of COVID-19 among students and staff members than did districts without mask requirements. Strengths of this investigation include the use of multiple analyses, and sensitivity analyses, with the protective effect of mask use holding across all analyses, including within districts that switched from no mask policy to any mask policy during the investigation period. Universal mask use, in coordination with other prevention strategies such as vaccination of students and staff members in K–12 schools, remains an important tool for preventing SARS-CoV-2 transmission (*8*).

On average, in the studied school districts, weekly COVID-19 incidences among students and staff members were higher than those in the surrounding communities; observed numbers of student and staff member cases were higher than expected based on community incidences for all mask policies. This highlights the potential for incidence within schools to be higher than that in communities in settings where community transmission levels are moderate to substantial and where the majority of students are unvaccinated. Expected numbers of school cases were calculated based on the assumption that cases in the wider community were as likely to be identified and reported as were those among students and staff members. Testing access was similar across the state, and there were no school-based testing programs in place during the investigation period.^†††† ^

The findings in this report are subject to at least five limitations. First, this was an ecologic study, and data on ventilation and other community and school-based prevention efforts were not available for inclusion in the analysis. However, surrounding community incidence was included in all analyses as a proxy for community-level factors (such as testing intensity) that could influence transmission or case identification that were not otherwise accounted for. Second, compliance with an existing mask policy was not directly observed or otherwise evaluated; however, noncompliance with mask policies would bias results toward the null. Third, quarantine rules differed for schools with and without mask requirements.^§§§§^ Students in schools with mask requirements were less likely to be quarantined than were their unmasked counterparts, also potentially biasing IRRs toward the null. Fourth, the pre- and postimplementation of mask policy analysis in a subset of 26 school districts could not separately investigate the impact of full and partial mask policies because of small sample sizes. Finally, data were obtained during a period of B.1.617.2 (Delta) variant predominance and might not be reflective of the current period of B.1.1.529 (Omicron) variant predominance; similar investigations could be beneficial as new variants arise.

This investigation indicates that school mask policies were associated with lower COVID-19 incidence in areas with moderate to substantial community transmission. Masks remain an important part of a multicomponent approach to preventing COVID-19 in K–12 settings, especially in communities with high COVID-19 community levels (*5*).

**Summary**

**What is already known about this topic?**

Masks are an important part of a multicomponent prevention strategy to limit transmission of SARS-CoV-2. Some school jurisdictions required masks in K–12 schools for fall 2021, while others did not.

**What is added by this report?**

In Arkansas during August–October 2021, districts with universal mask requirements had a 23% lower incidence of COVID-19 among staff members and students compared with districts without mask requirements.

**What are the implications for public health practice?**

Masks remain an important part of a multicomponent approach to prevent COVID-19 in K–12 settings, especially in communities with high levels of COVID-19.

## Footnotes

* Community attack rates were based on the weekly number of cases in the school district, minus the weekly number of cases among staff members or students during the same period. Denominators were calculated based on the population for each school district, minus the district student and staff member 2021–22 enrollment.

† Outdoor mask use requirements and mask requirements for student athletes who were actively participating in extracurricular sports were not considered when categorizing school district mask policies into full, partial, or none. Arkansas Department of Health guidance during the investigation period stated that outdoor masking was “not generally necessary” unless conditions were crowded.

§ Schools that serve blind, deaf, and incarcerated populations generally offer or require boarding, which might increase the risk for SARS-CoV-2 transmission. The year-round school district was excluded because its schedule was not comparable with other public school districts in Arkansas.

¶ Models used an autoregressive correlation structure of order 1 with a log population offset. The negative binomial generalized estimating equation model for the effect of mask policy (*A*) on COVID-19 incidence rates *(C^s^_ij_/N^s^_i_)* among students/staff members, adjusted for confounders is *ln(C^s^_ij_) = ln(N^s^_i_) + β_0 _+ β_1_A_1,i,j-1 _+ β_2_A_2,i,j-1 _+ β_3_J + β_4_lnR^c^_i,j-1 _+ β_5_V_i,j-1_ + β_6_L_i_* where school district *i* = 1, 2, 3, ..., 233; week *j* = 2, 3, ..., 8; observed cases in school district *i* and week *j* are given by *C^s^_ij_* ; community incidence rate in school district *i* and week *j* is given by *R^c^_ij_;* *N^s^_i_* is school district staff member and student population for school district *i*; *A_1_* and *A_2_* are full and partial mask policies; *V* is a vector representing categorical weekly vaccination coverage among students and staff members; *L* is a vector representing time-fixed categorical proportions of students receiving free or reduced-cost lunches during 2019.

** The expected number of cases for school district *i* during week *j* was estimated as follows: community cases in school district *i* and week *j* are given by *C^c^_ij_*; population estimates for the school district and community are given by *N^s^_ij_*, and *N^c^_ij_*, respectively. The expected number of cases for school district *i* and week *j* is given by *E^s^_ij_ = N^s^_ij_ (((C^c^_ij-1_ + C^c^_ij_)/2)/N^c^_ij_)*, where the community cases for a given week is a 2-week moving average of cases during the same week as the school cases and cases during the preceding week. The estimates of observed-to-expected numbers of cases by school district *i* and week *j* for modeling are given by *γ^s^_ij_ = C^s^_ij_/E^s^_ij_*. The base model is given by *ln(C^s^_ij_) = ln(E^s^_ij_) + β_0_ + β_1_A_1,i,j-1_ + β_2_A_2,i,j-1_ + β_3_J + β^5^V_i,j-1_ + β_6_L_i_.*

†† Twenty-six included districts represented urban and rural counties and were from each of Arkansas’ five public health regions, with an average enrollment of 1,130 students.

§§ School weeks were standardized to align the comparative interrupted time series (CITS) cut point (time zero) with the transition of mask policy from no masks required to a full or partial mask requirement. The cut point represents the week that any mask requirement was implemented, and the first weekly incidence under a mask requirement policy was measured during the following week. CITS first estimates baseline (i.e., before mask policy) linear trends in the dependent variable (weekly school-associated COVID-19 incidence) and separately, weekly community incidence. CITS then compares post-mask implementation policy period deviations for each group from those baseline trends. Consistent with models 1 and 2, an autoregressive (order 1) covariance structure was specified to incorporate 1-week lags between mask policy and COVID-19 incidence. Formally, the following regression specification was estimated using ordinary least-squares and standard errors: *y_t_ = β_0 _+ β_1_τ_t_ + β_2_Post_t _+ β_3_(τ_t _× Post_t_) + β_4_Treat + β_5_(τ_t _× Treat) + β_6_(Treat × Post_t_) + β_7_(τ_t _× Treat × Post_t_) + ε_t _*where *y_t_* is the COVID-19 infection rate per 100k during standardized week *τ_t_,* where *t *is an index for equally spaced time point. *Treat *is an indicator that is equal to 1 for the school (i.e., the treatment group) and zero for the community; *Post *is an indicator for post-mask policy implementation. The interaction term *(τ_t_ × Treat)* is a group-specific time trend that establishes separate baseline linear trends for school-associated and community COVID-19 incidence. The interaction term *(τ_t_ × Post_t_)* is a change in postintervention time trend that differentiates linear trends pre- and postimplementation of mask requirement policy. Finally, the interaction terms *(Treat × Post_t_)* provide estimates of changes in incidence rates between mask policy implementation weeks in the sample and baseline trends. These three interaction terms were used to determine whether pre- to postimplementation period changes in incidence rates differed for those who were directly affected by the policy change (i.e., staff members and students) and those who resided in the same community but were not directly affected by the mask policy.

¶¶ Some school boards based mask policies on locally available COVID-19 data. Policies were reevaluated weekly, monthly, or on an ad hoc basis, depending on the district.

*** For districts with mask policies that changed midweek, if the policy change occurred on Wednesday or later, the change was applied to the following week.

††† District-wide weekly COVID-19 non–school-associated (community) attack rates and student and staff member vaccination rates varied from week to week. Variables included in the analysis were based on the measurement the week before weekly student and staff member COVID-19 incidence (the outcome) was measured.

§§§ Analyses were stratified by vaccine eligibility because vaccination coverage data were not available at the school level.

¶¶¶ 45 C.F.R. part 46.102(l)(2), 21 C.F.R. part 56; 42 U.S.C. Sect. 241(d); 5 U.S.C.0 Sect.552a; 44 U.S.C. Sect. 3501 et seq.

**** COVID-19 incidence declined during the investigation period across the state, from a 7-day average high of 74.3 per 100,000 (substantial transmission = 50–99.99 cases per 100,000) on August 25, 2021, to 19.7 (moderate transmission = 10–49.99 cases per 100,000) on October 16, 2021. Vaccination rates across the state increased during the investigation period from 40% to 46.8%. https://covid.cdc.gov/covid-data-tracker/#datatracker-home

†††† Arkansas Department of Health recommended that exposed or symptomatic persons (including students and school staff members) get tested during the investigation period. However, there were no school surveillance testing programs nor test to stay programs in place during this time.
